# Novel Antibody-independent Method to Measure Complement Deposition on Bacteria

**DOI:** 10.21769/BioProtoc.4671

**Published:** 2023-05-05

**Authors:** Toska Wonfor, Shuxian Li, Maisem Laabei

**Affiliations:** Department of Life Sciences, University of Bath, Bath, UK

**Keywords:** Complement deposition, *Staphylococcus aureus*, Bacterial pathogenicity, Immune evasion, Flow cytometry

## Abstract

During infection, complement plays a critical role in inflammation, opsonisation, and destruction of microorganisms. This presents a challenge for pathogens such as*Staphylococcus aureus*to overcome when invading the host. Our current knowledge on the mechanisms that evolved to counteract and disable this system is limited by the molecular tools available. Present techniques utilise labelled complement-specific antibodies to detect deposition upon the bacterial surface, a method not compatible with pathogens such as*S. aureus*, which are equipped with immunoglobulin-binding proteins, Protein A and Sbi. This protocol uses a novel antibody-independent probe, derived from the C3 binding domain of staphylococcal protein Sbi, in combination with flow cytometry, to quantify complement deposition. Sbi-IV is biotinylated, and deposition is quantified with fluorophore-labelled streptavidin. This novel method allows observation of wild-type cells without the need to disrupt key immune modulating proteins, presenting the opportunity to analyse the complement evasion mechanism used by clinical isolates. Here, we describe a step-by-step protocol for the expression and purification of Sbi-IV protein, quantification and biotinylation of the probe, and finally, optimisation of flow cytometry to detect complement deposition using normal human serum (NHS) and both*Lactococcus lactis*and*S. aureus.*

## Background

*Staphylococcus aureus*has become a major global burden due to widespread infection within hospitals and communities (Murray et al., 2022). Due to the prevalence of antibiotic resistance, chronic-infections, and surgical complications, the demand to understand this pathogen and its relationship with the host has become critical (Tong et al., 2015). For a successful infection, pathogens must combat the first line of innate defence, the complement system. Complement is quick to identify pathogens, label them for efficient phagocytosis, and raise the alarm to other immune effectors of both the innate and adaptive systems (Noris and Remuzzi, 2013). For successful infection, pathogens must disable this pathway.*S. aureus*has a surprisingly large arsenal of complement-disabling proteins; however, due to restrictions in phenotypic assays, the details of virulence factors involved are not fully understood (Thammavongsa et al., 2015). The common method for observing complement activity/deposition utilises labelled antibodies that are detected by methods such as flow cytometry. In the case of*S. aureus*, this method is not possible due to the expression of two immunoglobulin binding proteins, Protein A (Spa) and Sbi, which bind indiscriminately to the Fc region of antibodies (Yang et al., 2018;Cruz et al., 2021). Previous attempts to bypass this issue used mutants unable to express IgG binding proteins (∆spa∆sbi); however, both proteins are important virulence factors in the defence against complement activation and are known to be expressed in >95% of clinical isolates.

In our study, we designed a novel probe utilising the C3 binding domain of Sbi (domain IV, seeFigure 1) and biotinylated it to allow recognition by fluorophore-labelled streptavidin (Clark et al., 2011;Wonfor et al., 2022). We show that it is able to bypass Spa/Sbi interference, and binding is specific to C3 deposition. This probe opens the door to analysing the immune evasion capacity of large collections of clinical isolates, allowing for a greater understanding of complement evasion at a population level.*Staphylococci*are not the only pathogens to bind IgG:*Streptococcus*has protein G, and*Peptostreptococcus*has protein L; therefore, this method may also enlighten the role of complement in other pathogen invasions (Sidorin and Solov'eva, 2011). Finally, Sbi-IV probe has the advantage of being very small in size. As a 11 kDa protein, in comparison to 150 kDa IgG antibody, the probe may have advantages outside immunoglobulin binding pathogens, and we envisage a role in in vivo diagnostics, such as detecting tissue inflammation.

**Figure 1.**
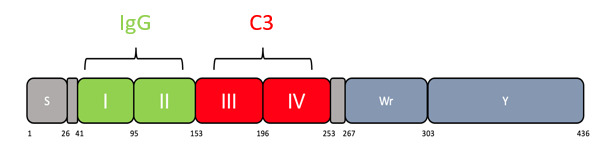
Scheme showing domains within Sbi protein. Residue numbers are shown below. Domains I and II bind IgG. Domains III and IV bind C3. Domain IV consists of residues 196–253. Additional residues added ensure stability and correct folding (Smith et al., 2012).

## Materials and Reagents

**General lab reagents**

1. Beakers, 250 mL
2. Eppendorf: 1.5 mL, 2 mL
3. Falcons: 15 mL, 50 mL
4. Flasks 2 L
5. Flea (magnetic stirring bar)
6. Foil
7. Petri dish/staining box
8. Syringe: 5 mL, 10 mL, 50 mL
9. Syringe needles
10. Centrifuge bottles compatible with specific floor centrifuge rotor (1 L, 50 mL)
11. Filter 0.45 μm (Thermo Fisher, Millipore, catalog number: SLHA033SS)
12. His-Trap column 1 mL (Fisher Scientific, Cytiva, catalog number: 10431065)
13. Microcuvettes 1.6 mL (Fisherbrand, catalog number: FB55147)
14. Size Exclusion Column HiLoad 16/600 Superdex 200 prep grade, 120 mL (Fisher Scientific, Cytivia, catalog number: 45002490)
15. Round bottom 96-well plate (Corning, catalog number: 3788)
16. Transfer pipette 5 mL sterile (Sigma, catalog number: HS206371C)
17. TurboBlot transfer pack (nitrocellulose membrane 0.2 μm) (Bio-Rad, catalog number: 1704158)
18. V-bottomed 96-well plate (Thermo Fisher, Nunc, catalog number: 249570)
19. BamHI-HF (NEB, catalog number: R3136S)
20. BL21 DE3 competent cells (NEB, catalog number: C2527I). Alternatively, make your own competent cells using CaCl_2_
21. GeneJET Miniprep kit (Thermo Fisher, catalog number: K0502)
22. HindIII-HF (NEB, catalog number: R3104S)
23. NovaBlue competent cells (Sigma, catalog number: 70181-3)
24. Phusion High-Fidelity PCR master mix (Thermo Fisher, catalog number: F351S)
25. T4 DNA ligase (NEB, catalog number: M0202S)
26. Wizard SV Gel and PCR clean up kit (Promega, catalog number: A9281)
27. Ampicillin (Fisher Scientific, catalog number: BP176025). Dilute powder in ddH_2_O to a final concentration of 100 mg/mL stock (store dilution at -20 °C)
28. Coomassie Brilliant Blue R-250 destain (Bio-Rad, catalog number: 1610438)
29. Coomassie Brilliant Blue R-250 stain (Bio-Rad, catalog number: 1610436)
30. Ethanol absolute (VWR, catalog number: 20821.330). Dilute to 20% with water
31. IPTG (Sigma, catalog number: 16758). Dilute powder in ddH_2_O to a final concentration of 0.5 M (store dilution at -20 °C)
32. Imidazole (Sigma, catalog number: I2399)
33. LB broth (Sigma, catalog number: L3022)
34. Laemmli sample buffer 4× (Bio-Rad, catalog number: 1610747)
35. mPAGE SDS running buffer powder (Sigma, catalog number: 20347927)
36. NaCl (Sigma, catalog number: S7653)
37. PageRuler Plus Prestained protein ladder (Thermo Fisher, catalog number: 26619)
38. Protease Inhibitor Cocktail Set VII (Sigma, Millipore Corp, catalog number: 539138)
39. SDS-PAGE precast gel 12% (Bio-Rad, catalog number: 4561043)
40. Trizma HCl (Sigma, catalog number: T3253)
41. Vivaspin 20 (5 kDa MWCO) (Sigma, catalog number: Z614580)
42. Vivaspin 500 (5 kDa MWCO) (Sigma, catalog number: Z614009)
43. BCA Protein Assay kit (Thermo Fisher, Pierce, catalog number: 23225)
44. BD Vacutainer^®^Clot Activator Tube (BD, catalog number: 367895)
45. ECL detection reagent (Cytivia, Amersham, catalog number: RPN2235)
46. Mini-PROTEAN TGX precast gel 4%–20% 10 wells (Bio-Rad, catalog number: 4561094)
47. Phosphate buffered saline (PBS) tablets (Thermo Fisher, Oxoid, catalog number: BR0014G)
48. Expression plasmid pQE30-Sbi-IV, available upon request
49. EZ-Link^TM^Sulfo-NHS-Biotinylation kit (Thermo Fisher, catalog number: 21425)
50. Skim milk powder (BD, Difco catalog number: 232100)
51. TBST powder (Sigma, catalog number: T9039)
52. Ultra-streptavidin-HRP (Thermo Scientific, catalog number: N504)
53. Barbituric acid (Sigma, catalog number: 185698)
54. Bovine serum albumin (BSA) (Sigma, Roche, catalog number: 03117332001)
55. Calcium chloride (CaCl_2_) (Sigma, catalog number: 223506)
56. Cell trace Far Red (Thermo Fisher, Invitrogen, catalog number: C34564)
57. FC loading tubes (5 mL polystyrene round-bottom test tube) (Fisher Scientific, Falcon, catalog number: 352052)
58. Fetal calf serum (FCS) (Thermo Fisher, Gibco, catalog number: A5256701)
59. Gelatin (from porcine skin) (Sigma, catalog number: G1890)
60. Compstatin (AMY-101) (MedChemExpress, catalog number HY-P1717)
61. Glucose (Sigma, catalog number: G8270)
62. Glass universal (for growing*S. aureus)*(Fisher Scientific, catalog number: 14823562)
63. M17 broth (Sigma, catalog number: 56156)
64. Magnesium chloride (MgCl_2_) (Sigma, catalog number: M8266)
65. Na-barbital (sodium 5,5-diethylbarbiturate) (Sigma, catalog number: B0500)
66. Streptavdidin-488 (Thermo Fisher, Invitrogen, catalog number: S32354)
67. Tryptic soy broth (Sigma, catalog number: T8907)
68. His A buffer (see Recipes)
69. His B buffer (see Recipes)
70. SEC buffer (see Recipes)
71. Milk block (see Recipes)
72. M17-G broth (see Recipes)
73. 10% FCS/PBS (see Recipes)
74. BSA/PBS (see Recipes)
75. Veronal buffer stock (VBS) (25 mM) (see Recipes)
76. GVB++ buffer (see Recipes)

## Equipment

1. Forceps (Fisher Scientific, catalog number: 15281209)
2. AKTA purifier 10 (Amersham Pharmacia Biotech)
3. Belly dancer/rocker (Fisherbrand, 3D Platform Rotator)
4. Bench centrifuge with compatible rotor for Eppendorfs capable of reaching speeds of 16,000*× g*
5. Centrifuge capable of speeds of at least 60,000*× g*and compatible with rotors for 50 mL and 1 L bottles (e.g., Beckman Avanti)
6. Centrifuge with buckets compatible for 96-well plates capable of reaching 3,400*× g*(Eppendorf, model: 5810 R)
7. Electrophoresis Power Supply (Cleaver Scientific, model: PowerPRO 300)
8. FACS CANTO Flow Cytometer (BD)
9. Falcon centrifuge capable of reaching 3,400*× g*(Eppendorf, model: 5810 R)
10. Freezer (-80 °C) for storage of serum and Sbi-IV protein
11. Gel imaging system (Azure Biosystem 400) for viewing chemiluminescence
12. Heat block reaching temperatures of 95 and 56 °C (Fisherbrand, Isotemp)
13. Magnetic stirrer (Stuart, model: US152)
14. NanoDrop/spectrophotometer (absorbance at 600 nm) (DeNovix, model: DS-11)
15. Protein electrophoresis tank (Bio-Rad, model: Mini-PROTEAN Tetra Cell)
16. Shaking incubator (capable of holding 2 L flasks and shaking at 200 rpm) set to 37 °C (e.g., Stuart SI500)
17. Sonicator (Soniprep 150 plus)
18. Spectrophotometer (absorbance at 562 nm) (Tecan, Sunrise)
19. Static incubator, set to 37 °C (Fisher Scientific, HERATherm, catalog number: 10744262)
20. Thermocycler (Applied Biosystem, SimpliAmp)
21. Trans-Blot Turbo Transfer System (Bio-Rad)
22. Western Blot Roller (Thermo Fisher, catalog number: 84747)

## Software

1. BD FACSDiva (BD) for flow cytometer
2. FlowJo^TM^v10 software (BD Life Sciences) (www.flowjo.com)
3. GraphPad Prism 8 (Dotmatics) (www.graphpad.com)
4. Unicorn 5.32 (Cytiva) for AKTA

## Procedure

- **Cloning, protein expression, and purification of Sbi-IV**
  1. Using the Phusion High-Fidelity PCR master mix kit, amplify the IV domain of Sbi protein (amino acids V198-A266) with*S. aureus*Mu50 genomic DNA and primers: Sbi-IV forward (CGGGATCCGTTTCAATTGAAAAAGCAATC) and Sbi-IV reverse (CCCAAGCTTTCATTACGCCACTTTCTTTTCAGC), using 60 °C annealing temperature and 15 s extension time.
  2. Thermocycling conditions following manufacturer’s protocol: initial denaturation: 98 °C, 30 s; 30 cycles of (denaturation: 98 °C, 5 s; annealing: 60 °C, 30 s; extension 72 °C, 15 s); final extension: 72 °C, 5 min; 4 °C ∞.
*Notes:*
    - *Our pQE30-Sbi-IV plasmid is available upon request.*
    - *pQE30 contains a His-tag. If using an alternative expression plasmid, please include a His-tag to the cloned region during PCR.*
  3. Digest 1 μg of both the PCR product and pQE30 plasmid with restriction enzyme BamHI-HF and HindIII-HF. Incubate for 4 h at 37 °C using a thermocycler.
*Note: We recommend performing the plasmid digest in quadruplicate to improve yield after gel extraction.*
  4. Gel-extract the digested plasmid and clean up digested PCR product following the Wizard SV Gel and PCR clean up kit protocol. Quantify the product using a NanoDrop.
  5. Ligate the plasmid and PCR products using a molar ratio of 3:1 with NEB T4 DNA ligase enzyme. Incubate at 16 °C overnight using a thermocycler.
  6. The next morning, transform competent cells (NovaBlue) with 1 μL of ligation following the suggested heat-shock protocol. Grow on LB agar containing ampicillin (100 μg/mL).
  7. Miniprep the plasmid using GeneJET miniprep kit.
  8. Transform 10 ng of plasmid into BL21 DE3 competent cells.
*Note: Always use freshly transformed cells for protein expression for maximum efficiency.*
  9. Inoculate 2 × 20 mL of LB broth with a colony of BL21 DE3*E. coli*containing pQE30-Sbi-IV plasmid. Add 100 μg/mL ampicillin. Grow cultures overnight at 37 °C with shaking at 180 rpm.
  10. The next morning, pour the overnight culture into two 2 L flasks containing 500 mL of LB. Add 100 μg/mL ampicillin and grow culture at 37 °C with shaking until it reaches an OD_600nm_between 0.5 and 0.7.
  11. Add 0.5 mM IPTG (500 μL of 0.5 M stock) to each flask and grow for a further 3 h.
  12. Pour the cultures into four 500 mL centrifuge bottles and centrifuge at 4,000*× g*for 10 min at 4 °C.
  13. Resuspend the pellet in 40 mL of 4 °C His A buffer.
    - Resuspend the first pellet in 40 mL of buffer, then move that resuspended solution into the next bottle. All four pellets should be resuspended in the same 40 mL solution.
    - Move solution into a 50 mL Falcon.
  14. Repeat the centrifugation step and discard the supernatant.
*Notes:*
    - *At this step, the pellet can be frozen and stored at -20 °C (or -80 °C long term).*
    - *To continue, thaw the pellet on ice for 30 min.*
  15. Resuspend the pellet in 30 mL of fresh His A buffer and add 300 μL of Protease Inhibitor Cocktail Set VII.
  16. Lyse the cells by sonicating in 10 s bursts for 10 min at 80% amplitude.
*Notes:*
    - *Fill a beaker with ice and place the falcon within. Ensure that the pellet solution is fully surrounded by ice. Refill ice between sonication bursts if it melts away.*
    - *Use a large sonicator tip.*
    - *Always use ear protection.*
  17. Pour solution into a 50 mL centrifuge bottle (use a second tube with the same weight of water for balancing) and centrifuge the lysed solution at 60,000*× g*for 30 min at 4 °C.
*Notes:*
    - *Balance with equivalent weight water.*
    - *DO NOT discard the supernatant!*
  18. Filter the supernatant through a 0.45 μm filter using a 50 mL syringe. Filter solution into a clean 50 mL Falcon.
  19. Prepare the AKTA by washing the lines with water, followed by attaching the His-column.
*Notes:*
    - *Reduce the flow speed to 1 mL/min.*
    - *Loosen the storage caps on either end of the column. Remove top cap and attach the AKTA line. Ensure liquid fills the inlet valve of the column before screwing in. This prevents bubbles from forming within the column. Release the bottom screw and wait for a drop of liquid to form at the bottom of the column before attaching to the AKTA.*
    - *Once column is attached, ensure flow speed is limited to column capacity, i.e., 1 mL/min. Exceeding this speed could damage the column.*
  20. Wash the column with five column volumes of water.
*Note: Run for 5 min at 1 mL/min.*
  21. Place the intake line A1 into His A buffer and line B1 into His B buffer and wash the column with buffer A for 10 column volumes (10 min).
*Note: Line numbers may differ between AKTAs.*
  22. Load the cassette with thirty 2 mL collection tubes ready for elution.
*Note: The required number of tubes may also vary.*
  23. Once the AKTA is prepared, load the filter-sterilised sample onto the AKTA via the injection valve (you may require a large loading line).
  24. Run a pre-set program for His-Tag purification using the Unicorn software.
*Notes:*
    - *This will wash the sample through the column with His A buffer allowing His-tag binding. Once the sample is washed through, it will incrementally switch from buffer A to buffer B containing a high concentration of imidazole. This will displace the His-tag and elute the bound proteins.*
    - *Always double-check the flow speed and elution volume.*
  25. Label collected fractions by number and store in the fridge.
*Note: Note which fraction numbers showed a high peak at 280 nm. This can be used to predict which fractions contain your protein.*
  26. Wash the His-column with 10 column volumes of water, followed by 5 column volumes of 20% ethanol.
  27. Remove the column and store in the fridge for future use.
    - Continue running the AKTA at 1 mL/min.
    - While it is running, remove column carefully by first disconnecting the bottom screw. Replace with the storage cap. When disconnecting the top, allow a drop of liquid to fill the top of the inlet value before attaching the cap to prevent air from getting trapped within the column.
  28. Run alternating fractions on an SDS-PAGE gel.
    - Take 15 μL from each even number fraction. Alternatively, run the fractions indicated by a 280 nm peak.
    - Add 5 μL of 4× Laemmli sample buffer and heat samples at 95 °C for 5 min using a pre-heated heat block.
    - Load the samples onto the precast gel. Include 5 μL of protein ladder in the end well.
  29. Run gel at 200 V for 60 min (or until loading dye reaches bottom of the gel).
  30. Stain the gel by placing into a Petri dish and pouring over Coomassie Brilliant Blue R-250 stain (enough to cover the gel). Place on a rocker for 1 h.
  31. To destain, pour out the used Coomassie and pour in a similar volume of Coomassie Brilliant Blue R-250 destain. Incubate for 15 min on a rocker. Pour out and replace with fresh destain and incubate overnight.
*Note: Coomassie stain contains hazardous chemicals and must be disposed of carefully.*
  32. View gel on a light box or gel dock.
  33. Identify the recombinant protein using the protein ladder and the protein predicted molecular weight. It should be a very thick and prominent band. Pool together all fractions containing target protein, as indicated by the gel.
*Note: Include those with visible contaminant proteins, if continuing with a second round of purification.*
  34. Concentrate the pooled fractions using Vivaspin 20 centrifugal concentrator. Note the MWCO size to ensure no loss of protein (for Sbi-IV, use MWCO 5 kDa).
*Notes:*
    - *Load sample into the top of column.*
    - *Centrifuge at 3,400 × g until sample volume drops below the 5 mL line (approximately 10 min).*
    - *Collect sample from the top of the spin column and discard the flowthrough.*
  35. To remove aggregate proteins from sample, load into a 15 mL Falcon and centrifuge at 10,000*× g*for 10 min.
*Note: Collect supernatant using a needle attachment on syringe. This ensures that you collect every drop.*
  36. Prepare AKTA and attach Size Exclusion Column (SEC). For further details on the delicate procedure of attaching and detaching columns, follow the manufacturer’s protocol.
    - Place line A1 in SEC buffer and line B in ddH_2_O water.
    - Set AKTA flow (with water) to 1 mL/min.
    - Attach SEC adaptor line and wait for it to fill with solution.
    - When a drop of liquid is released on the end, carefully screw into top of the SEC column. Always allow the inlet valve to fill with solution before attaching.
    - Detach the buffer plug and screw in the bottom column line.
    - Wash with water for 1.5 column volumes, followed by the same volume of SEC buffer.
  37. Wash the 5 mL loading line by injecting solution using a 25 mL syringe. Wash with ethanol, then water, followed by SEC buffer before loading sample.
  38. Load sample onto AKTA and run prepared SEC program.
  39. Use the 280 nm absorbance to predict fractions containing the recombinant protein, which should show a clear peak.
*Note: The elution fraction can also be predicted by the protein size and capacity of the column. As our protein was very small, we can assume the protein will be eluted in the final fractions.*
  40. Run samples on gel that showed a peak on 280 nm graph (or where the protein was predicted to elute) to confirm protein location (seeFigure 2).
    **Figure 2.**
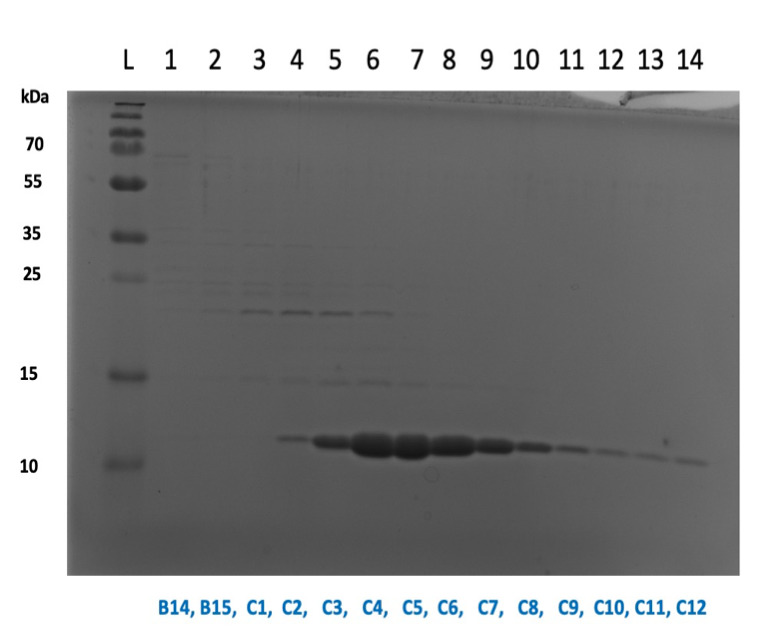
SDS-PAGE gel showing collected aliquots following purification. Here, aliquots C4–C12 were pooled together and concentrated. Store collected fractions at -20 °C after confirming fractions on gel. Thaw samples slowly on ice, and pool protein fractions together. Be sure to only combine those containing*pure*protein with no contaminant bands. Concentrate using Vivaspin 20 & 500 concentration columns (centrifuge at 4,000*× g*and 4 °C). Concentrate until sample reaches a volume between 250 and 500 μL.
- **Protein concentration determination**
  1. Perform BCA using Pierce^TM^BCA Protein Assay kit.
  2. Follow the manufacturer’s protocol. All reagents required are included in the kit. In addition, a spectrophotometer/microplate reader capable of reading absorbance at 562 nm is needed.
  3. Compare sample absorbance to standard curve to calculate concentration of sample. You may have to dilute the sample 1:10 or more to fit the curve.
*Note: We collected 0.5 mL of protein at 5.2 mg/mL.*
- **Biotinylation**
  1. Perform biotinylation using EZ-Link^TM^Sulfo-NHS-Biotinylation kit following the manufacturer’s detailed protocol.
*Note: This kit biotinylates the protein using lysine residues. This can impact protein function if lysines are exposed within functional sites.*
  2. Defrost the recombinant protein on ice. Using the concentration calculated by BCA and protein molecular weight in Da, calculate the required concentration of biotin. Make sure the sample volume falls between 0.5 and 2.0 mL.
    - Calculate millimoles of Sulfo-NHS-LC-Biotin to add to the reaction for a 20-fold molar excess:

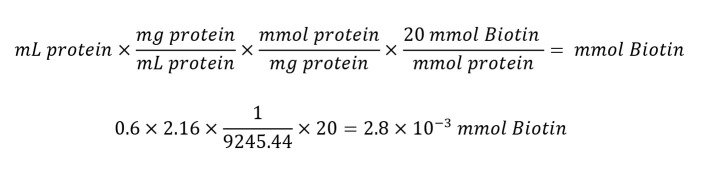

    - Calculate microliters of 10 mM Sulfo-NHS-LC-Biotin to add to the reaction:

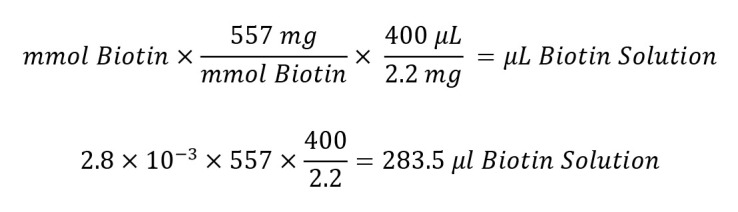

  3. Perform buffer exchange of protein sample using one of the desalting columns included in the kit. This is to replace the SEC buffer with PBS.
  4. Dilute 2.2 mg of Sulfo-NHS-LC-Biotin in 400 μL of ultrapure water and add the*calculated*volume of biotin solution to your protein. Incubate on ice for 2 h.
  5. Repeat buffer exchange to remove excess biotin. Aliquot the sample into small volumes (10 μL) to reduce freeze–thawing cycles.
- **Western blot**
  Western blots are useful tools to confirm if the biotinylation of the protein was successful.
  1. Begin by running 100 ng of biotinylated and non-biotinylated protein on an SDS-PAGE gel.
    - Prepare 15 μL of 100 ng biotinylated and non-biotinylated protein. Dilute to required concentration using PBS.
    - Add 5 μL of 4× Laemmli sample buffer to each sample.
    - Heat sample at 95 °C for 5 min using a heat block.
    - Prepare Mini-PROTEAN TGX precast gel (4%–20%) by removing the tab at the bottom and fixing the gel in the gasket. Fill buffer chamber with mPAGE SDS running buffer.
    - Load the full 20 μL of sample on the precast gel.
    - Add 5 μL of protein ladder to an end well.
    - Perform electrophoresis with 150 V for approximately 1 h (or until the blue loading dye is visible at the very bottom of the gel).
  2. Transfer proteins onto a 0.2 μm nitrocellulose membrane using Trans-Blot Turbo Transfer System (any other transfer system will also work).
    - Open the Trans-blot turbo cassette and assemble the blotting sandwich (use Bio-Rad’s*Quick-start guide*to assemble correctly).
    - On the base of the cassette, place the*bottom*membrane using forceps. Place your gel carefully over the membrane (we recommend trimming off the comb and any excess/unused wells before placing). Once the gel touches the membrane, do not adjust it.
    - Place the*top*membrane over the gel and close the cassette.
    - Place the cassette into the Trans-blot machine and run on*turbo mini gel 4*pre-set program. This will only take 7 min.
  3. Using forceps, remove the upper transfer layers and SDS-PAGE gel to reveal the membrane.
    *Note: If transferred correctly, the dye from the ladder should be visible on the membrane*.
  4. Carefully, with forceps, place the membrane in a clean Petri dish and immediately pour over 10% milk block.
*Note: Be careful to never let the membrane dry. Have the milk block prepared before transfer.*
  5. Place dish on a rocker for 1 h (at room temperature).
  6. Wash the membrane three times with TBST.
    Pour off the milk block and pour on enough TBST to cover the membrane. Leave on rocker for 5 min and pour out. Repeat three times.
  7. Pour on 5% milk block with 1:20,000 dilution of ultra-streptavidin-HRP. Incubate on the rocker for 1 h (at room temperature).
  8. Repeat the membrane wash three times.
  9. Add ECL detection reagent and image the gel.
    - In a Falcon, mix 1 mL of ECL reagent A with 1 mL of ECL reagent B.
    - With forceps, move the membrane into a clean Petri dish.
    - Using a pipette, cover the membrane with the mixed solution. Cover with foil and incubate for 30 s.
    - Move the membrane onto some clear film and use a roller to push away excess ECL solution.
    - Place on a gel imager and read*chemiluminescence*. Use the*auto image*function ~30 s.
*Note: This can be optimised with different protein and antibody dilutions and exposure times. If biotinylation has been successful, a visible band should only be visible on the biotinylated protein, as shown inFigure 3.*
      **Figure 3.**
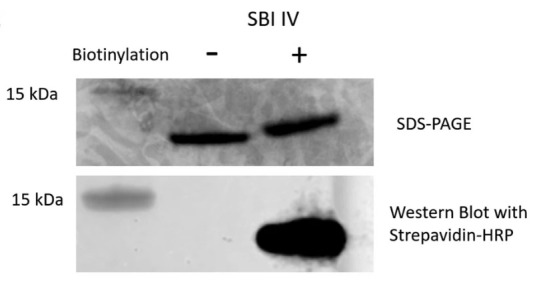
SDS-PAGE and western blot analysis of biotinylated and nonbiotinylated Sbi-IV. Figure taken from Wonfor et al. (2022).
- **Serum collection**
*Note: Collection and experimental use of serum requires ethical approval by your institution.*
  1. Using a qualified phlebotomist, collect blood from**at least**eight people (5 × 10 mL from each individual). Blood must be collected in Clot Activator tubes to ensure proper clotting.
    Collect written consent forms for each donor.
  2. Incubate the blood collection tubes containing blood for 30 min at room temperature so that it is well clotted.
    - Write down the time of blood collection for each individual so that all tubes are left to clot for the same amount of time.
    - After clotting, place the tubes on ice.
    - Continue all remaining steps on ice or at 4 °C.
  3. Centrifuge clotted tubes at 700*× g*for 7 min at 4 °C.
  4. Using a transfer pipette, move all the sera (clear yellow liquid) from the blood tubes into a clean 50 mL Falcon (on ice). Be careful not to lift any of the clotted blood (red pellet). Pool all the sera from one individual into the same Falcon.
  5. Spin down the individual sera at 700*× g*for 7 min at 4 °C.
  6. Take the supernatant and pool into one autoclaved 100 mL beaker on ice.
*Notes:*
    - *It is* ***essential*** *to avoid picking up any blood (red pellet). Leave a little serum behind to make this easier.*
    - *Be sure to autoclave the beaker with a flea (magnetic stirring bar).*
  7. In a 4 °C room, set up an aliquoting station. Place tube storage boxes open on ice and fill with open Eppendorf tubes.
  8. Briefly mix the pooled serum**from all donors**on a magnetic stirrer.
*Note: Proper mixing is essential to ensure all aliquots are uniform in composition.*
  9. In the 4 °C room, begin aliquoting the serum into tubes. We recommend aliquoting in 50, 100, and 250 μL aliquots, working as quickly as possible.
  10. Immediately after aliquoting, store them at -80 °C.
    *Note: The protocol below shows the conditions for observing complement deposition upon*L. lactis. L. lactis*has no virulence factors to evade complement, which makes it a useful tool for optimizing the conditions for the new probe. This protocol was optimized with*L. lactis*strain MG1363.*
- **Complement deposition and labelling upon*L. lactis*with Sbi-IV**
  1. Set up an overnight culture of*L. lactis.*Inoculate 5 mL of M17-G with a single colony of*L. lactis*and incubate at 30 °C overnight (static).
  2. The following morning, thaw an aliquot of NHS slowly in a bucket of ice. Thaw a 5 mL aliquot of GVB++ buffer in a beaker of hot water for 30 min, and then place on ice.
  3. Prepare a 96-well V-bottomed plate by adding 200 μL of 10% FCS/PBS to all wells. Incubate for 30 min on the bench.
  4. Wash the wells three times by filling with 200 μL of PBS. Tap the plate face down on blue roll to dry.
  5. Centrifuge 1 mL of overnight*L. lactis*culture at 16,000*× g*for 5 min. Remove the supernatant and resuspend in 1 mL of PBS buffer. Repeat the centrifugation to wash away any residual culture media and discard supernatant. Finally, resuspend in 1 mL of fresh PBS.
  6. Normalise the bacteria to an OD_600nm_= 1.
    - Read absorbance of 1 mL by diluting 1:10 in a cuvette.
    - Calculate the volume of cells required for OD_600nm_= 1. The target volume will depend on how many samples you wish to run. 50 μL is required per sample (add ~100 μL excess for gating).

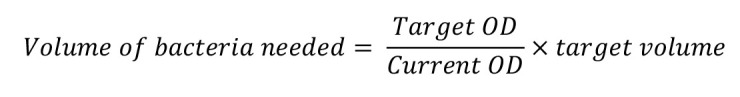

    - Using the calculation above, add the*volume needed*into a fresh Eppendorf.
    - Keep an aliquot of unstained bacteria on ice for initial gating.
  7. To stain with cell trace Far Red, add 1 μL of cell trace per 1 mL of OD_600nm_= 1 bacteria (to a final concentration of 1 μM). Cover with foil and incubate for 20 min at 37 °C with shaking at 180 rpm. From this point on, keep stained bacteria covered with foil.
  8. To remove excess stain, either:
    - (For small volumes, <500 μL) add 3× volume 3% BSA/PBS and incubate on bench for 10 min.
    - (For large volumes, >500 μL) centrifuge for 5 min. Discard the supernatant. Resuspend the pellet in 1 mL of 3% BSA/PBS. Incubate on the bench for 10 min.
  9. Centrifuge bacteria for 5 min at 16,000*× g*and resuspend pellet in*target*volume of GVB++. Bacteria are now stained and normalised to OD = 1.
  10. Make a 2× stock of serum (NHS) by diluting in GVB++ buffer. As before, the volume required is dependent on the number of samples, where 50 μL is required per sample.
*Notes:*
    - *NHS concentration is generally described as % and the concentration required will need optimizing. We recommend beginning with a gradient of 2% to 20%. For a*no serum control*just add GVB++ buffer.*
    - *Only dilute NHS when you are ready to add to the plate. Once diluted, use immediately.*
  11. Add 50 μL of diluted NHS and 50 μL of stained bacteria to the coated V-bottomed well plate. Cover with foil and incubate for 30 min at 37 °C (static).
*Note: Keep plate covered with foil for all remaining incubation steps.*
  12. Centrifuge the plate at 3,400*× g*for 7 min (at room temperature) (if possible, set the deceleration speed to 6). Discard the supernatant gently (careful not to disturb the pellet) and resuspend each well in 100 μL of 1% BSA/PBS. Repeat the centrifugation and gently discard the supernatant.
  13. Dilute primary probe in 1% BSA/PBS. Prepare enough for 100 μL per sample.
*Note: Dilutions vary depending on the probe/antibody. For Sbi-IV, we used a final concentration of 175 μM, but we would recommend performing your own optimization using various dilutions of your purified stock (i.e., 1:250, 1:500, 1:1,000, and 1:2,000). Determine which concentration gives a high geometric mean with minimal background on negative controls.*
  14. Resuspend the cells in 100 μL of probe solution and incubate on the bench for 45 min.
  15. Repeat centrifugation and wash as described in step 12.
  16. Dilute secondary probe (with conjugated fluorophore) in 1% BSA/PBS. Make enough for 100 μL per sample (for streptavidin-488, dilute 1:1,000). Keep diluted stock covered with foil until ready to use.
  17. Resuspend bacteria in 100 μL of secondary probe solution and incubate on the bench for 45 min.
  18. Repeat centrifugation and wash from step 12, then resuspend for a final time in 100 μL of PBS. Keep plate covered with foil and carry over to the flow cytometer.
- **Complement deposition and labelling upon*S. aureus*with Sbi-IV**
  *Note: Follow the protocol as described above with the adaptations listed below. This protocol was optimized with*S. aureus*strain JE2).*
  1. Set up an overnight culture of*S. aureus.*Inoculate 2 mL of tryptic soy broth with a single colony of*S. aureus*and incubate at 37 °C with shaking at 180 rpm overnight.
  2. The following morning, subculture the overnight culture 1:200, either in 5 mL (25 μL of culture) or 50 mL (250 μL), and grow with shaking at 37 °C to OD_600nm_= 0.5–0.6.
*Notes:*
    - *Two hours is enough for*S. aureus*strain JE2. The time will vary between different strains, but usually takes 2–3 h. For slower strains (such as TW20), we recommended changing the subculture to 1:100 or 1:50.*
    - *We recommend growing*S. aureus*in either 25 mL glass universals or glass flasks.*
  3. When normalising (step F6 above), for*S. aureus*we recommend normalising to OD_600nm_= 2.
  4. To stain (step F7 above), add 2 μL of 1 mM cell trace per 1 mL of OD_600nm_= 2*S. aureus*(2 μM final concentration).
  Controls that can be used in optimisation and analysis of complement deposition on bacteria using Sbi-IV to identify any non-specific binding in the absence of C3 deposition:
  1. No NHS = GVB buffer only
  2. CP40-NHS = Incubate 2× NHS stock with 50 μM compstatin (AMY-101). Incubate on ice for 30 min before mixing with bacteria.
  3. HI-NHS = Heat 2× NHS stock at 56 °C using a heat block for 30 min. Cool briefly on ice before mixing with bacteria.
  4. C3 depleted NHS (purchased) = dilute in GVB to 20% (= 2× stock). Add as normal.
    *Note: The flow cytometry protocol written here can also be used with commercial antibodies to study complement deposition on non-immunoglobulin binding pathogens (such as*L. lactis*). This can include antibodies targeting both C9 as well as C3. i.e., rabbit anti-human C3d (Dako), or mouse anti-human C9 (aE11, Abcam). Note that you will need compatible fluorophore-labelled secondary antibody.*
- **Setting up FACS CANTO**
  Read sample using FACS CANTO (BD) with lasers capable of reading wavelengths of 488 nm and 633 nm. Count 20,000 events.
  1. Prepare the flow cytometer for reading samples.
    - Switch on FACS Canto.
    - Launch FACSDiva software.
    - Run the program Fluidics Start Up.
*Note: This will wash the system and allow time for the lasers to warm up.*
  2. Acquire data from samples.
    - Transfer 100 μL of sample from a 96-well plate to an FC loading tube.
    - Push lever to the left and insert tube onto the probe (Figure 2). Release the lever returning it to its starting position.
*Note: The lever base should sit comfortably under the tube; if not, push it on a little further (as shown inFigure 4below).*
      **Figure 4.**
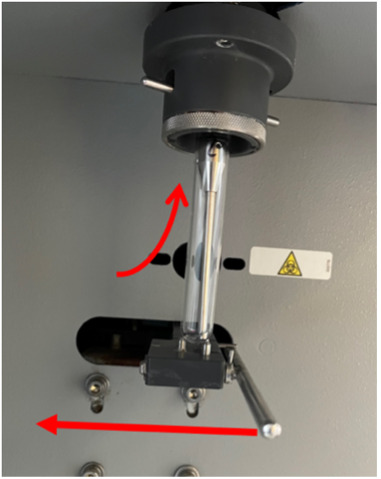
Flow cytometry sample insertion
    - Click Acquire Data and wait approximately 10 s (this allows the number of events per second to settle), before clicking Record Data. This will automatically stop acquisition when the threshold*events to record*is reached (set this to 20,000 events, as shown inFigure 5below).
      **Figure 5.**
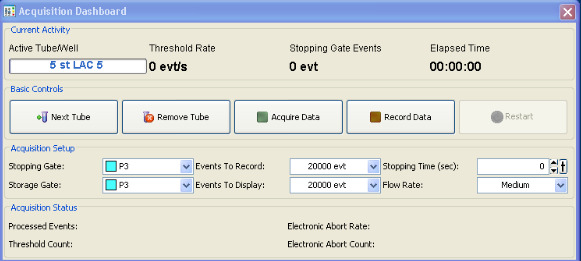
Flow cytometry data acquisition
    - Click Remove tube and wait for the progress box to pop up. Push the lever**all the way to the left**, remove the tube, and then release the lever.
*Note: This automatically triggers the FACS CANTO to wash the probe. If you do not move the lever properly, an error will pop up.*
    - Click Next tube when ready to acquire the next sample.
  3. Prepare a worksheet for collecting samples. On the right-hand side, open a Global Worksheet.
*Note: Setting up workbooks and gating for flow cytometry can be complicated and very easy to manipulate incorrectly. We highly recommend setting this up with an experienced technician, to ensure the gating is performed accurately. These are the graphs we recommend:*
    - SSC vs. FSC (side vs. forward scatter, to gate bacteria).
    - FSC-H vs. FSC-A (forward area vs. height).
    - Histogram for APC-A (633 nm) to gate stained bacteria.
    - Histogram for Alexa Fluor 488-A (488 nm) to observe probe.
    - APC-A vs. Alexa Fluor 488-A.
  4. Initial setup requires adjusting the cytometer parameters under Cytometer (Figure 6). Select FSC-A, FSC-H, SSC-A, SSC-H, 488-A, and APC-A. Make sure to select Log for all four parameters.
    **Figure 6.**
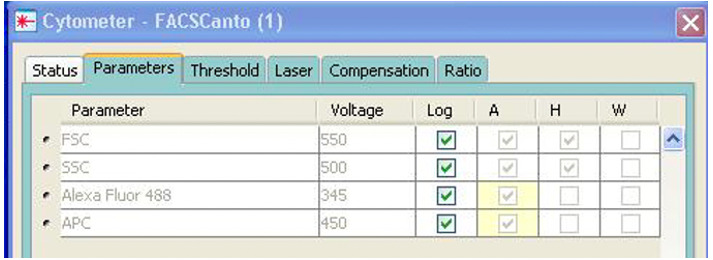
Adjusting cytometer parameters
  5. Begin gating by loading 100 μL of**unstained**bacteria into an FC loading tube (Figure 7). Click Acquire Data.
    *Note: You do not need to click Record data while adjusting the voltages and gating. Only Record experiment samples*.
    - View particles on the FSC vs. SSC. Adjust the voltages of FSC and SSC until the bacterial population is visible and close to centre of the graph.
    - FSC shifts the particles right or left. Increasing FSC voltage shifts to the right, and lowering it shifts the scatter to the left.
    - SSC voltage shifts the particles up or down. Increasing SSC voltage shifts the scatter up, and lowering the voltage shifts the scatter down.
    - Apply a box gate around the cluster of cells. This will be gate P1.
      **Figure 7.**
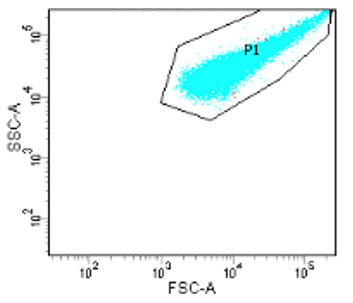
Gating of bacterial sample
    - Perform doublet discrimination: examining the FSC-A vs. FSC-H, apply a second gate (P2) that fits tightly to the bottom-right edge of the cluster (Figure 8). This will remove doublets.
*Note: When two particles pass the laser at the same time, the flow cytometer designates this as a single event; performing doublet discrimination increases accuracy of your analysis.*
      **Figure 8.**
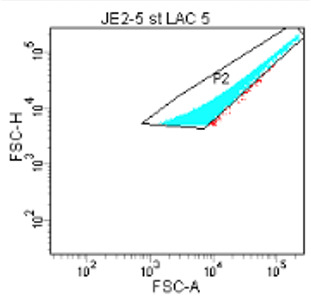
Removing doublet events
  6. Remove the sample and load 100 μL of**stained**bacteria (Figure 9).
    - View the histogram for APC-A and adjust the APC voltage until the peak for stained bacteria is visible.
    - Increasing the APC voltage shifts the visible peak to the right. Lowering the voltage shifts to the left.
    - On the histogram for APC, add a gate around the visible peak with stained bacteria (Gate P3). Ensure there is no peak within this gate when running a sample of unstained bacteria.
  7. Finally, run a positive control sample (i.e.,*L. lactis*+ 10% serum) to adjust the peak for 488. Biotinylated Sbi-IV will be used to detect complement deposition.
    **Figure 9.**
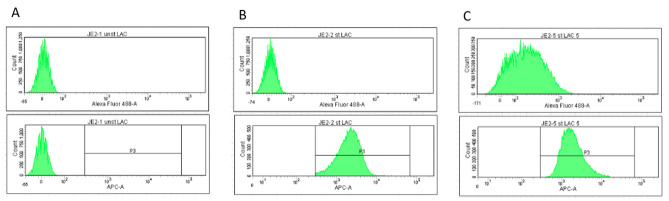
Screenshots showing histograms for 488-A (top; biotinylated Sbi-IV) and APC-A (bottom; bacteria). A) Unstained bacteria, 0% serum. B) Stained bacteria, no serum. C) Stained bacteria + 5% serum. P3 gate is essential to ensure counting only occurs on stained bacteria.
  8. The final gate is applied to APC-A vs. 488-A. This separates the graph into quadrants based upon the histograms viewed above (Figure 10).
    - Q1: Particles visible here are stained with APC but have no labelled Sbi-IV or antibodies bound (no 488 signal).
    - Q2: Particles here are both stained AND have labelled Sbi-IV or antibodies deposited.
    - Q3: Particles here are unstained and have no labelled Sbi-IV or antibodies (possible contamination).
    - Q4: Particles here are not stained but do have labelled Sbi-IV or antibodies deposited upon them.
*Note: For Q4, this result would strongly suggest improper staining of cells.*
      **Figure 10.**
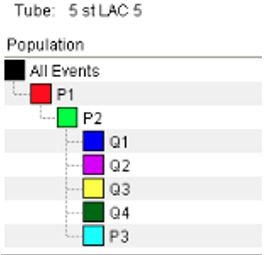
Final gating of workbook. P3 may be above Q’s. Ensure the stopping and storage gates are set to P3.
  9. These settings and gates may have to be adjusted with each new experiment; therefore, always begin by running an unstained and stained control before collecting data.
  10. Save the data by clicking on the active workbook and click Export, followed by FCS files. Select FCS 3.0 and continue. This will save the samples in the correct format for analysis in FlowJo.
  11. Clean the cytometer before shutting down. Follow the routine recommended by your technicians.
    - Wash probe by running 10% bleach for 5 min (click Acquire, do not click Record). Repeat with water for 5 min. Make sure the FC sample tube is at least half full with water before running Fluidics Shutdown.
    - Close the FACS Diva software and turn of the machine by pushing the green button.
  12. Analysing the data in FlowJo v10 Software (Figure 11):
    - Open FlowJo software and drag and drop FCS files into the open box.
    - As all the samples were gated, and counted events were restricted to only those within the P1, P2, and P3 gate, you do not need to gate within FlowJo. However, we recommend adding the Q1–Q4 gate to assist in visualising the results.
    - Double-click on a sample. Adjust the x- and y-axis to APC-A vs. 488-A.
    - Add the gate as close to the position originally placed in FACS DIVA.
*Note: This gate is not used for quantitative analysis; only observation.*
    - To analyse geometric mean of 488, click on the Statistics box on the bottom of the pop-up box. Click the Sigma+, which will open another box. Select Geometric mean on the left, and Alexa Fluor 488-A on the right, followed by Add. Close both boxes.
    - Highlight all four Q gates + geometric mean (shift click), then drag and drop onto All samples in the box above. This will apply the same gate to all samples.
      **Figure 11.**
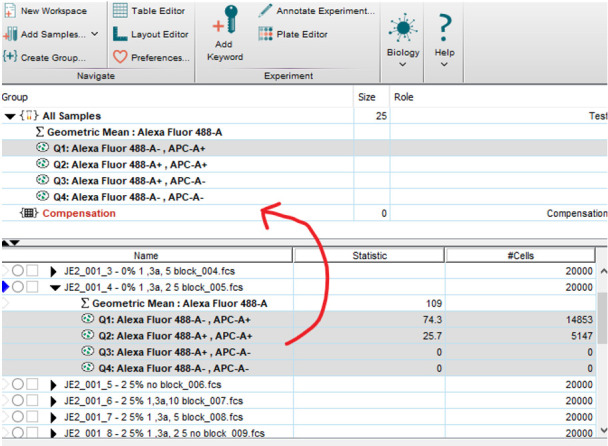
Data acquisition using FlowJo
  13. The geometric mean value observed under Statistic is the number used to plot geometric mean in a graph. We performed this in GraphPad.
  14. Scatters/histograms can also be observed by dragging and dropping into the Layout editor.

## Recipes

1. **His A buffer (500 mL)**
  50 mM Tris (Trizma HCl)
  150 mM NaCl
  20 mM imidazole
  ddH_2_O to final volume
  Adjust to pH 7.4
  Filter-sterilise with 0.22 μm filter and autoclave
  Store at 4 °C
2. **His B buffer (500 mL)**
  50 mM Tris (Trizma HCl)
  150 mM NaCl
  500 mM imidazole
  ddH_2_O to final volume
  Adjust to pH 7.4
  Filter-sterilise with 0.22 μm filter and autoclave
  Store at 4 °C
3. **SEC buffer (500 mL)**
  20 mM Tris (Trizma HCl)
  150 mM NaCl
  ddH_2_O to final volume
  Adjust to pH 7.4
  Filter-sterilise with 0.22 μm filter and autoclave
  Store at 4 °C
4. **Milk block**
  For 10% block, dilute 1 g of skim milk powder in 10 mL of TBST buffer
  For 5% block, dilute 0.5 g of skim milk powder in 10 mL of TBST buffer
  Shake well to dissolve
5. **M17-G broth**
  - Prepare M17 broth by dissolving in ddH_2_O and send to autoclave.
  - Make 10% glucose by diluting in ddH_2_O, and filter-sterilise with 0.45 μm filter.
  - Add 10% glucose stock to a final concentration of 1%.
6. **10% FCS/PBS**
  - Dilute FCS in PBS to a final concentration of 10% FCS.
  - Filter-sterilise using 0.45 μm filter.
  - Store at 4 °C.
7. **BSA/PBS**
  - Add BSA powder to PBS to a final concentration of 1% or 3% (1 g per 100 mL of PBS for 1%, 3 g per 100 mL of PBS for 3%).
  - Filter-sterilise using 0.45 μm filter.
  - Store at 4 °C.
8. **Veronal buffer stock (VBS) (25 mM) (500 mL)**
  NaCl (720 mM)
  Na-barbital (9.0 mM)
  Barbituric acid (15.5 mM). Dissolve powder in hot water first
  ddH_2_O to final volume
  Adjust to pH 7.35
  Filter-sterilise using 0.45 μm filter
  Aliquot in 10 mL/50 mL batches
  Store at -20 °C
9. **GVB++ buffer (500 mL)**
  5 mM veronal buffer (1:5 dilution of VBS); thaw in hot water for 20 min
  0.1% (w/v) gelatin. Dissolve powder in hot water first
  1 mM MgCl_2_
  0.15 mM CaCl_2_
  ddH_2_O to final volume
  Filter-sterilise using 0.45 μm filter
  Aliquot in 5 mL batches
  Store at -20 °C
